# Standing in Others' Shoes: Empathy and Positional Behavior

**DOI:** 10.3389/fpsyg.2019.02226

**Published:** 2019-10-17

**Authors:** Alpaslan Akay, Gökhan Karabulut, Bilge Terzioğlu

**Affiliations:** ^1^Department of Economics, University of Gothenburg, Gothenburg, Sweden; ^2^Institute of Labor Economics (IZA), Bonn, Germany; ^3^Department of Economics, Universidad Antonio de Nebrija, Madrid, Spain; ^4^Department of Economics, Istanbul University, Istanbul, Turkey

**Keywords:** dispositional empathy, survey experiments, positional concerns, utility, subjective well-being

## Abstract

Studies show that people are concerned with other people's consumption position in a varying degree with respect to the type of goods consumed and individual characteristics. Using both survey experiments and a large survey of subjective well-being (SWB) dataset, this paper aims to investigate the association between the degree of empathic capacity and positional concerns for consumption items involving pleasure and pain. The paper exploits both empathy quotient (EQ) and interpersonal reactivity index (IRI) measures of empathic capacity, i.e., dispositional empathy, which are sufficient measures capturing affective and cognitive aspects of empathy. Positional concerns are identified directly using a series of stated choice experiments and indirectly using the SWB approach. The main result of the paper is that positional concerns vary substantially with the levels of empathic capacity. Both EQ and IRI are found to be *positively* associated with positional concerns for “goods” (e.g., after-tax income, market value of a luxury car), reflecting a degree of self-regarded feelings and behavior to reduce personal distress, and *negatively* associated with positional concerns for “bads” (e.g., working hours and poverty rates), reflecting a degree of other-regarding feelings and behavior. The results are robust with respect to various checks including statistical specifications, reference groups, and omitted variables (e.g., prosocial behavior and competitivity) that could bias the results.

JEL Codes: C90; D63.

        “As we have no immediate experience of what other men feel,

        we can form no idea of the manner in which they are affected,

        but by conceiving what we ourselves should feel in the like situation.”

                                                                                         Adam Smith, *The Theory of Moral Sentiments*

## Introduction

Empathy is one of the basic processes that make us connect with other people's feelings, emotions, and experiences (Batson, 1987, 1991; Eisenberg and Miller, 1987; Eisenberg et al., 1994; Brandstätter, 2000; Keum and Shin, 2016). It is most often considered to be the capacity or skill of “projecting yourself into what you observe” (Davis, 1980; Batson, 1991; de Waal, 2008, 2012). In *The Theory of Moral Sentiments* (Smith, 1759), Adam Smith extensively discussed the importance of empathy^1^—as quoted above—in particular how it is associated with the other-regarding and self-interested behaviors in human life. Indeed, studies in fields ranging from neurobiology to psychology have already accumulated a bulk of evidence that empathy has evolved to predict other people's behavior, feelings, and experiences of pleasure and pain (e.g., Batson, 1991; Baron-Cohen and Wheelwright, 2004; Singer et al., 2006; de Waal, 2008; Cronin, 2012; Klimecki et al., 2016)^2^. Thus, it is not surprising that behavioral economists give attention to how empathy is related to prosocial behavior including altruism, cooperation, and fairness considerations (e.g., Edele et al., 2013; Klimecki et al., 2016). How we emotionally connect with and react to other people's feelings, emotions, experiences of pleasure and pain might also be one of the building blocks of *processes of social comparisons* (“positional” or “status” concerns) with others (Tesser et al., 1988; Tesser, 1991; Brandstätter, 2000). The present paper aims to investigate how people's degree of empathic capacity relates to their positional concerns with respect to consumption goods associated with experiences of pleasure and pain.

Positional concerns have long been discussed by various scholars including Adam Smith, Karl Marx, and Veblen, and the topic is currently attracting substantial empirical interest among social psychologists and economists (Senik, 2004; Ferrer-i-Carbonell, 2005; Clark et al., 2008; Akay et al., 2013). These concerns imply that individuals' utility is related not only to their own absolute level of consumption but also to their level of consumption relative to that of relevant others, i.e., their reference or comparison groups (Clark and Senik, 2010). One consequence of these comparisons is the negative externality causing personal distress and large welfare loss (Clark et al., 2008). The literature has identified important impacts of these externalities on economic issues ranging from labor supply and migration to optimal taxation (e.g., Neumark and Postlewaite, 1998; Aronsson and Johansson-Stenmann, 2014; Akay et al., 2017). However, little is known about the fundamental processes underlying positional behavior. Recently, another strand in the literature has focused on how positional concerns relate to contextual factors, individual socio-demographic characteristics, and trait-like constructs including emotions, personality characteristics, and empathy (e.g., Buunk et al., 1990; Tesser, 1991; VanderZee et al., 1996; Brandstätter, 2000; White et al., 2006; Akay and Martinsson, 2011, 2019; Blázquez Cuesta and Budría, 2015; Budria and Ferrer-i-Carbonell, 2018). Drawing on this literature, to best of our knowledge first time, this study takes a comprehensive approach to investigate the relationship between the levels of “dispositional” or “trait” empathy and positional concerns. To this end, we use both a series of tailor-made survey experiments (e.g., Solnick and Hemenway, 2005; Carlsson et al., 2007) dealing with an array of goods and the subjective well-being (SWB) approach that is based on a large survey of SWB and empathy-related information (e.g., Ferrer-i-Carbonell, 2005; Akay and Martinsson, 2011).

Researchers seem to agree that empathy operates as an affective (“empathic emotions”) and cognitive (“perspective taking”) *reflection process* that helps the person connect to other people's feelings and experiences (Batson, 1991; Tesser, 1991; Chopik et al., 2017). The empathic reflection process is also expected to operate when people compare their levels of consumption with those of other people (Tesser et al., 1988; Tesser, 1991; Brandstätter, 2000; Batson et al., 2002; de Waal, 2008, 2012). This process may function as a source of information about the experience of others and might lead to substantial heterogeneity in the degree of positional concerns, which might also differ by the type of good under consideration, e.g., whether it is “a luxury car” or “poverty experience” (Tesser et al., 1988; Brandstätter, 2000). An increase in the consumption level of a “good^3^”—a consumption item that is associated with pleasure or utility—by an “average” relevant other person in an individual's reference group is expected to increase the personal distress and reduce the individual's well-being (Clark et al., 2008). Yet someone with higher empathic capacity might become more distressed than other people as this person identifies the pleasure experience of others better. This person may try to selfishly seek a better consumption position to get a similar pleasant experience. Thus, we predict that a higher level of empathy might trigger a higher degree of self-regarding behavior and competition for a better consumption position for a “good” (Zillmann and Cantor, 1977; Batson, 1987; Lanzetta and Englis, 1989; Batson et al., 1991; de Waal, 2008; Cronin, 2012). Yet, the empathic reflection process regarding other people's level of consumption of a “bad”—a consumption item associated with pain or disutility—might lead to completely different feelings and reactions. In this case, empathic reflection on the feelings and experiences of others might trigger “compassion” or “pity.” Thus, a person with higher empathic capacity is expected to act altruistically by competing less for a better position in the case of consumption items signaling suffering of others (Batson et al., 1991; de Waal, 2008). Thus, we expect that greater empathic capacity is negatively related to positional concerns about items involving pain or disutility.

To investigate the associations between the levels of empathic capacity and positional concerns, we use two approaches that are often used to identify positional concerns. The first approach is based on a stated choice experiment with a hypothetical scenario where respondents make a series of decisions about the consumption levels of their “future relative” compared to “strangers” living in the same society or country, i.e., their reference group (Carlsson et al., 2007). The survey experiments identify the heterogeneity in positional concerns directly on individual utilities for a series of consumption items and elicit the long-form of empathy quotient (EQ) to capture the degree of empathic capacity (e.g., Baron-Cohen and Wheelwright, 2004; Edele et al., 2013). The second approach is based on SWB information in which the degree of positional concern is indirectly identified using the absolute and relative level of consumption of individuals (Ferrer-i-Carbonell, 2005; Luttmer, 2005). The SWB dataset used is obtained from the General Social Survey (GSS), which is high-quality representative cross-sectional data (Einolf, 2008). In this approach, the interpersonal reactivity index (IRI) by Davis (1980, 1983) is used as a measure of empathy. It is obtained from the National Altruism Study Module supplied as a part of GSS for the years 2002 and 2004. Our extensive investigation shows that two alternative approaches with two measures of empathy produce strikingly similar results. Highly in line with the expectations, both the EQ and IRI measure of empathy are *positively* related with the degree of positional concerns for “goods” implying self-regarded feelings and behavior and *negatively* related with the degree of positional concerns for “bads” implying other-regarded feelings and behavior. We find that these results are highly robust with respect to control variables, functional form, reference groups, estimators, and proxies for the potential omitted variables (e.g., prosocial behavior, competitivity, envy, and self-esteem).

The remaining part of the paper is organized as follows. Next section describes our survey experiment, i.e., the setup, descriptive and conditional results, and a detailed robustness analysis. Section Evidence from Subjective Well-Being Data gives the evidence from the SWB approach, where we present the dataset, econometric specifications, results, and robustness analysis. Finally, section Concluding Discussions concludes the paper.

## Evidence from Survey Experiments

### Setup

#### Procedure

The survey experiment consisted of two parts^4^. First, our experiment assistants presented a script with a scenario and a set of hypothetical binary choice questions to 307 randomly recruited respondents^5^. They were asked to imagine “a future relative,” for example a grandchild who is going to live two generations from now. The choice situations in the survey experiment involved a series of decisions about the best society/country for the imaginary grandchild to live in. In the second part of the survey experiment, the respondents completed a questionnaire aimed to elicit (*i*) socio-demographic and -economic characteristics, (*ii*) psychological measures including empathy measures obtained using 60 questions of the EQ, personality characteristics (Big-5), self-esteem, and emotions, and (*iii*) attitudes to prosocial behavior, competitivity, and inequality. That is, the respondents first made experimental decisions and then answered a series of neutral questions including questions about socio-demographic characteristics such as age, gender, university department, and family characteristics. Finally, the EQ questionnaire was distributed. To control for a possible trend (due to, e.g., fatigue, conformity, or alienation) across the repeated answers by the respondents, the decisions were arranged in six different orders of goods. Our empirical model specifications are also controlled for the order of questionnaire dummies to allow this sort of confounders.

#### Utilities

In the first part of our survey experiment, the respondents were asked to decide which society, Society (A) or (B), they would like their imaginary grandchild to live in. Both societies consist of “strangers” and differ only in terms of the grandchild's *absolute* and *relative* amount of consumption. The experimental assistants carefully described the hypothetical scenario and the example choice situation (see Appendix A). To measure individual-specific positional concerns for a good *g*, we begin with a utility function *U*^*g*^(*Y*^*g*^, *Y*^*g*^ − *Y*^*gR*^) involving absolute level of consumption *Y*^*g*^ and relative level of consumption *Y*^*g*^ − *Y*^*gR*^ of good *g*. The functional form of the utility function is chosen to be linear for simplicity:

(1) $$\begin{array}{r} U^{g}\left(Y^{g},Y^{g}-Y^{gR}\right)\;=\left(1-\lambda^{g}\right)Y^{g}+\lambda^{g}\left(Y^{g}-Y^{gR}\right). \end{array}$$

In Equation (1), λ^*g*^ is the parameter capturing the degree of positional concerns with respect to good *g*. λ^*g*^ can be interpreted as the fraction of marginal utility due to an increase in relative consumption of good *g*. Thus, a higher level of λ^*g*^ implies that individuals show a higher level of positional concern with respect to good *g*. The main aim of the experiment was to identify the *mean degree of positional concerns* (MDPC hereafter) for each good *g*. We used relatively large reference groups *R*, which consisted of “strangers” in a society or country. The design aims to exclude potential confounding emotions stemming from the socio-cultural and genetic proximity between individuals and the people in their reference groups (see, e.g., Tesser et al., 1988 and Brandstätter, 2000 for discussions on the empathic reflection process in relation to liked and disliked particular others).

Having specified the utility function for the whole population, we generate a series of binary choice situations with different combinations of absolute and relative levels of consumption for the future grandchild and other people in each society/country. Appendix A presents the outlines of the hypothetical scenario and the example choice situation for after-tax income/month. The income levels were chosen so that they *implicitly* involve a degree of positional concern once Society (B) is chosen. Imagine that the respondent is indifferent between choosing Society (A) and Society (B). Then we can write

(2) $$\begin{array}{r} \left(1-\lambda^{g}\right)Y_{A}^{g}+\lambda^{g}\left(Y_{A}^{g}-Y_{A}^{gR}\right)=\left(1-\lambda^{g}\right)Y_{B}^{g}+\lambda^{g}\left(Y_{B}^{g}-Y_{B}^{gR}\right), \end{array}$$

and implementing the income levels given in Appendix A, we obtain

(3) $$\begin{array}{r} \lambda^{g}=\frac{Y_{A}^{g}-Y_{B}^{g}}{Y_{A}^{gR}-Y_{B}^{gR}}=\frac{2,000-1,800}{2,500-1,500}=0.20. \end{array}$$

This figure implies that the respondent's degree of positional concern should be at least 0.20 ( λ^*g*^ > 0.20) once Society (B) is chosen. To find the marginal interval of a respondent's degree of positional concerns, we ask repeated binary questions involving combinations of absolute and relative levels of consumption corresponding to an increasing set of implicit degree of positionality as 0.25, 0.50, and 0.75 (see Appendix B.1 for three binary choice situations in case of the after-tax income/month experiment). That is, the experiment identifies the “marginal” interval of positionality by identifying the question at which the respondent switches from choosing Society (B) to Society (A) for each individual and good *g*. We experiment with several goods that differ in terms of the feeling and attitudes they are expected to trigger. The list of goods, choice situations, absolute and relative consumption levels, and corresponding implicit degrees of positional concerns are presented in Appendix B.2.

### Measuring Empathy

Several strategies to measure empathy are suggested in the literature (e.g., Davis, 1980, 1983; Baron-Cohen and Wheelwright, 2004; Gerdes et al., 2010; Neumann et al., 2015). Our measure of empathy is the empathy quotient (EQ), which is based on a set of survey items (Baron-Cohen and Wheelwright, 2004). EQ is found to be a sufficient measure to identify both affective and cognitive dimensions of dispositional empathy (Lawrence et al., 2004; Edele et al., 2013). The measure mainly identifies the “trait” or “skill” dimension of empathy, with a higher level implying a higher level of dispositional empathic capacity (see Baron-Cohen and Wheelwright, 2004 for a detailed account of the measure).

EQ is based on 60 survey items (see Appendix C.1 for the full set of expressions/statements). Yet, only 40 items are actually used to construct the scale; the only purpose of the rest of the items is to distract attention and prevent answers that trigger social desirability and individual alienation. The EQ scale is generated as follows: Each statement/expression in the inventory is responded to on a four-point scale, i.e., “*strongly disagree*,” “*disagree*,” “*agree*,” and “*strongly agree*.” There are two groups of items. In the first group (numbered 1, 6, 19, 22, 25, 26, 35, 36, 37, 38, 41, 42, 43, 44, 52, 54, 55, 57, 58, 59, and 60 in Appendix C.1), respondents score 2 empathy points if they choose “*strongly agree*” and 1 point of empathy if they choose “*agree*.” In the second group (numbered 2, 3, 5, 7, 9, 13, 16, 17, 20, 23, 24, 30, 31, 33, 40, 45, 47, 51, 53, and 56 in Appendix C.1), respondents score 2 empathy points if they choose “*strongly disagree*” and 1 point if they choose “*disagree*.” The rest of the questions are scored as 0 as they merely serve as controls (numbered 2, 3, 5, 7, 9, 13, 16, 17, 20, 23, 24, 30, 31, 33, 40, 45, 47, 51, 53, and 56 in Appendix C.1). The Cronbach's alpha for the forty-items used in the construction of EQ scale is 0.84 which is very high and highly in line with the previous studies [e.g., alpha reported in Baron-Cohen and Wheelwright, 2004 is about 0.91].

In our experiment, we obtained 267 fully completed EQ questionnaires. Eliminating respondents with at least one missing answer and those with inconsistent answers^6^ reduced the sample to 224 observations for after-tax income/month, 214 for the market value of a luxury car, 231 for weekly working hours and poverty rates (%) experiments. The distribution of EQ scores is highly symmetric with a mean (median) value of 47.8 (47) and a standard deviation of 11.01. The minimum EQ score is found to be 16 and the maximum 76. The distribution of EQ is highly similar to that of studies using EQ (see, e.g., Baron-Cohen and Wheelwright, 2004; Edele et al., 2013).

### Unconditional Results

#### Overall MDPC

As the first step of our analysis, we present the share of positional respondents—unconditional estimates of MDPC—split by goods and choice situations in Column I of Table 1. Fifty-two percent of the respondents chose the positional alternative, Society (B), for after-tax income/month. Sixty-one percent of respondents are positional when the implicit degree of positional concerns is 0.25, while the proportion decreases to 52 and 43% as the implicit degree is increased to 0.50 and 0.75 in the subsequent choice situations. The percentage of positional respondents is 56% for the market value of a luxury car, which is slightly higher than that for after-tax income/month. Yet the difference in shares of positional choice across these two goods is not statistically significant at conventional levels. The next two items are working hours/week and poverty rates (%). Only 39% percent of the respondents chose the positional alternative for working hours/week. The share of positional choices is significantly smaller than that for after-tax income/month (Mann-Whitney-*U*-test *p* < 0.001). The share of positional choice is 45% for the poverty rates (%). The positional behavior regarding poverty rates (%) is also lower than that for after-tax income/month and the market value of a luxury car (Mann-Whitney-*U*-test *p* = 0.043 for after-tax income/month and *p* = 0.002 for the market value of a luxury car). Overall, the unconditional MDPC estimates are about 0.39–0.56, which are highly similar to the values in previous studies that used a similar sample and experimental design (c.f. Akay et al., 2013) and in samples from other countries (c.f. Carlsson et al., 2007).

**Table 1:** Unconditional results.

	**Share of choosing positional alternative**	**Share of positional choice among**		**Mann-Whitney-*U*-Test (*p*-values)**
		**Low dispositonal empathy (EQ < Median)**	**High dispositional empathy (EQ > Median)**	
	**I**	**II**	**III**	**IV**
**After tax income/month (in TRY)**	0.521	0.446	0.578	0.004
Society A				
Society B(1)	0.612	0.545	0.658	0.074
Society B(2)	0.520	0.446	0.575	0.046
Society B(3)	0.432	0.347	0.500	0.016
**Market value of a car (in TRY)**	0.558	0.511	0.582	0.069
Society A				
Society B(1)	0.642	0.589	0.676	0.090
Society B(2)	0.576	0.522	0.604	0.222
Society B(3)	0.457	0.422	0.464	0.540
**Working hours (week/hours)**	0.386	0.446	0.354	0.013
Society A				
Society B(1)	0.501	0.565	0.471	0.077
Society B(2)	0.363	0.435	0.321	0.071
Society B(3)	0.295	0.337	0.269	0.130
**Poverty rates (%)**	0.451	0.526	0.435	0.012
Society A				
Society B(1)	0.555	0.603	0.504	0.058
Society B(2)	0.484	0.532	0.448	0.093
Society B(3)	0.399	0.444	0.352	0.068

*Authors' own calculations from the experimental data*.

*TRY is the new Turkish Lira. EQ is the empathy quotient (Baron-Cohen and Wheelwright, 2004)*.

#### Heterogeneity in MDPC by EQ

The remaining columns of Table 1 present the descriptive results of our survey experiment for the different levels of EQ. Columns II and III show the share of positional choice for each good and choice situation split by low and high EQ levels. We identify individuals with a higher and lower level of empathic capacity using the median level of EQ = 47 as threshold. The unconditional MDPC is higher among people with a higher empathic capacity for after-tax income/month and the market value of a luxury car, i.e., “goods.” The share of positional choice is statistically different among people with lower and higher EQ for both after-tax income/month and the market value of a luxury car. The Mann-Whitney-*U*-test *p*-values are presented in the final column of Table 1 (Column IV). In most cases, the *p*-values suggest significant differences at conventional levels.

The next two items involve individual pain or disutility, i.e., “bads.” While people who work longer hours earn more and might obtain a better income position, they also suffer as working longer hours involves disutility (Knabe and Rätzel, 2010). The unconditional results suggest that a higher EQ level relates to a lower share of respondents with positional concern with respect to working hours/week. The difference in the share of respondents with positional preferences across the levels of EQ is statistically significant at the conventional levels, *p* = 0.013. Finally, we focus on the poverty rates (%), which is a public “bad” and expected to involve a high degree of suffering. Indeed, the poverty rate can be considered as an overall measure for the degree at which the people in the society suffer. In line with our predictions, the respondents with higher empathic capacity show a lower level of positional concern. The Mann-Whitney-*U*-test suggests that the difference in share of positional choice across the EQ levels is highly significant with *p* = 0.012.

#### Detailed Results by Choice Situations

**Figure 1:**
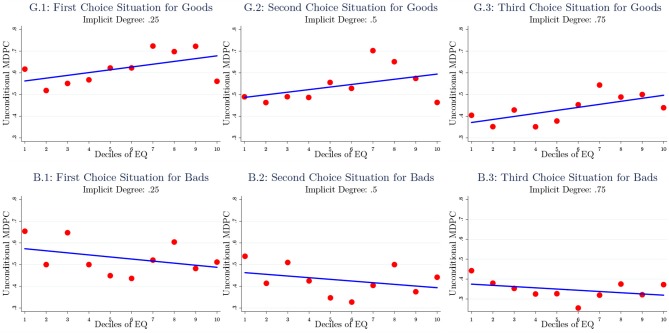
The unconditional relationship between EQ and share of positional choice. The figure displays unconditional relationship between the deciles of EQ (horizontal axis) and the unconditional MDPC (vertical axis). (G.1–G.3) Merge the income and car experiments, and (B.1–B.3) merge the working hours and poverty rates experiments. The relationship is presented for three choice situations with 0.25, 0.5, and 0.75 implicit degrees of positional concerns. The lines represent the linear regression based on the underlying data.

Figure 1 presents unconditional results to give further ideas about the relationship between the levels of empathic capacity and positional concerns. First, to obtain higher degrees of freedom, we merge the experimental data from the after-tax income/month and the market value of a luxury car experiments as “goods,” and working hours/week and poverty rates (%) as “bads.” Figure 1 presents the relationship by splitting for the three choice situations for both “goods” and “bads.” Along the horizontal axis are the 10 deciles of the EQ distribution and on the vertical axis we present unconditional estimates of MDPC for each decile. We also show the linear regression line (using the underlying data−10 observations in this case) to illustrate the strength of the unconditional relationship between the level of empathic capacity and positional concerns. A clear pattern emerges, i.e., the relationship is positive for the “goods” (G.1–G.3) and negative for the “bads” (B.1–B.3) for each choice situation. The strength of the relationship is similar across the choice situations, which change only with respect to the underlying implicit degree of positional concern (0.25, 0.5, and 0.75).

### Econometric Analysis

#### Model Specification

To identify the association between EQ and positional concerns conditional on a set of individual characteristics, we estimate a series of interval regressions as we measure positionality in an interval for each individual and good. The estimation model reads:

(4) $$\begin{array}{r} {{\tilde{\theta }}_{i}^{g}=\text{X}}^{\prime }\beta +\alpha EQ_{i}+P^{\prime }\phi {\;+\;\epsilon }_{i}^{g}, \end{array}$$

where ${\tilde{\theta }}_{i}^{g}\;$is the latent marginal positionality interval with upper ${\tilde{\theta }}_{i}^{g\left(lower\right)}$ and ${\tilde{\theta }}_{i}^{g\left(upper\right)}$ boundaries for each individual *i* and good *g*. The interval regression in model (4) allows for a set of observed characteristics, **X**, including age, gender, household income (in seven category dummies), household size, number of siblings, health status (four dummies from “very poor” health to “very good” health), department of the university (dummies for economics, psychology, and law), and six order-effect dummies. β is the corresponding vector of parameters. The key variable in this study is our empathy measure EQ and the parameter of interest is α. The baseline model specification is based on the logarithm of EQ, which allows a degree of flexibility in the relationship between EQ and positional concerns. In our robustness checks, we also estimate models with alternative functional forms including the standardized levels of EQ and a dummy variable indicating high empathic capacity. The model specification (4) is estimated using the maximum likelihood estimator, which assumes the normal distribution for the good-specific error terms $\epsilon_{i}^{g}$.

#### Stochastic Specifications

The experimental setup in this study does not allow us to make causal interpretations of the relationship between EQ and positional concerns. That is, the results should be interpreted as correlations. Clearly, EQ might be correlated with the good-specific error terms $\epsilon_{i}^{g}$. Equation (4) might have omitted variables or positional concerns might determine people's empathy level, e.g., reverse causality. In both cases, our results might be substantially biased. In this paper, we assume that dispositional empathy is a trait exogenously given to individuals. Therefore, the variation in the levels of empathy is assumed to be temporal due to contextual factors. Nevertheless, there might still be some variables that are persistently correlated with both the level of empathy and positional concerns, leading to omitted variables bias.

Our approach to alleviate the omitted variables bias is to allow our model specifications for some proxies that are potentially correlated with EQ and error terms $\epsilon_{i}^{g}$. We suggest three important proxies that could capture potential omitted factors. The first is *overall well-being*, measured using life satisfaction—a measure of SWB. Respondents with higher life satisfaction may engage more in social life and helping behavior and experience less positional concern (Diener and Larsen, 1984; see also Dolan et al., 2008 for a general review of the determinants of SWB). Second, we allow our regressions for a measure of *inequality aversion*, which might be one of the factors underlying non-positional behavior and may correlate with EQ (Fehr and Schmidt, 1999)^7^. The third set of proxies involves *personality* characteristics measured using the so-called five factor model (Big-5, *extraversion, agreeableness, conscientiousness, neuroticism*, and *openness-to-experience*)^8^. These characteristics are considered to measure non-cognitive skills, e.g., memory, social skills, and motivation, and have been found to be hard-wired constructs as they are stable after adolescence (McCrae and John, 1992; Cobb-Clark and Schurer, 2013). We then include these proxies in matrix **P**, and ϕ is the vector of corresponding parameters. In our robustness analysis, we will include several other proxies, e.g., prosocial behavior and competitivity as well as emotions (e.g., envy) and self-esteem, to tease out potential variables driving the relationship.

### Conditional Results

#### Baseline

**Figure 2:**
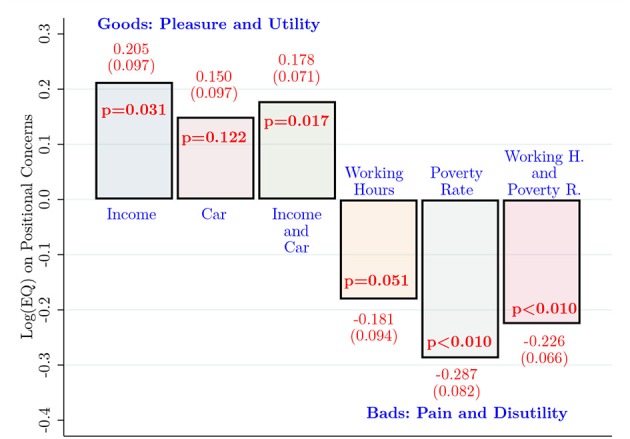
Baseline results: interval regressions. The bars present parameter estimates of log EQ on positional concerns obtained from the baseline model specification (4). The dependent variable is the marginal positionality interval for each respondent. The interval regressions control for the full set of control variables: age, gender, household income after tax (in seven income categories), a dummy indicating whether the respondent lives with parents, university department (economics, psychology, or law), household size, overall well-being (five dummies), inequality aversion, Big-5 personality traits (extraversion, agreeableness, conscientiousness, neuroticism, and openness-to-experience). Robust standard errors are presented in parentheses under the parameter estimates. *P*-values (*p*) are presented inside the bars.

Our baseline model specification is an interval regression as presented in Equation (4). The maximum likelihood estimation of the model specification is summarized in Figure 2^9^. The full estimation results are not presented as the focus of our paper is on the relationship between EQ and positional concerns^10^. We are mainly interested in the sign, significance, and relative magnitude of EQ on positional concerns across goods. Conditional on the full set of socio-demographic and -economic variables (see the note in Figure 2), overall well-being, inequality aversion, and Big-5 personality traits, the logarithm of EQ is positively and significantly associated (*p* = 0.031) with positional concerns regarding after-tax income/month. The parameter estimate of EQ on positional concerns regarding the market value of a luxury car is also positive, but the magnitude of it is lower than that of after-tax income/month and it is not estimated with lower precision (*p* = 0.122). The positive parameter estimates of EQ on positional concerns for “goods” are highly in line with our predictions. In the third bar of the first group of goods (pleasure and utility), we present results by combining the experimental data from the after-tax income/month and the market value of luxury car experiments. The parameter estimate of EQ is positive and statistically significant on positional concerns (*p* = 0.017).

We now turn our attention to consumption items that involve pain or disutility. First, we estimate the baseline specification (4) for positional concerns regarding working hours/week. The parameter estimate of EQ is negative and statistically significant at conventional levels, *p* = 0.051. That is, a higher level of empathic capacity is associated with a lower level of positional concern regarding longer working hours/week. Second, we estimate the baseline model specification with the data from the poverty rates (%) experiment. In line with the predictions, the parameter estimate of EQ is negative, large in magnitude, and highly statistically significant, *p* < 0.01. The final bar combines these two items into one data set. Overall, a higher level of empathy is associated with a lower level of positional concerns with respect to “bads.”

#### Heterogeneity

On average, the baseline results suggest a significant association between empathic capacity and positional concerns, yet the sign and magnitude of the association differ across goods. An important direction of analysis is to predict the MDPC across the levels of EQ conditional on the full set of individual characteristics. To this end, the estimated baseline interval regression is exploited to predict conditional MDPC for specific levels of EQ. MDPC is calculated for a more flexible functional form of EQ by adding the quadratic term in the baseline. Prediction is obtained by holding all control variables fixed at their mean values except EQ. Then, the MDPC and standard errors of predictions are calculated using several values of EQ from 20 to 80 in 5-point steps. Confidence intervals based on normal distribution are calculated to identify whether the degree of heterogeneity in MCPC is statistically significant across the levels of EQ.

**Figure 3:**
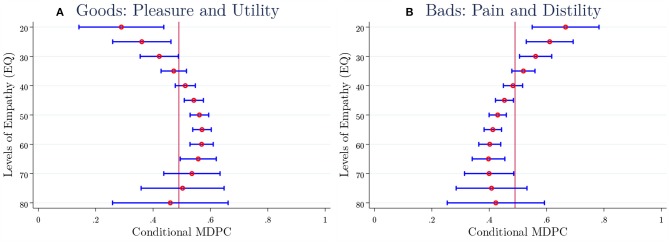
Heterogeneity of Conditional MDPC by EQ Levels. The panels present predicted conditional MDPC (horizontal axis) from the baseline interval regression (4), which uses a quadratic function of empathy (EQ). The levels of EQ are given along the vertical axis. The dependent variable is the marginal positionality interval for each respondent. The interval regressions control for the full set of controls (see Figure 2). **(A)** Combines data for the after-tax income/month and market value of car experiments while **(B)** combines data from working hours/week and poverty rates (%) experiments. The horizontal lines represent 90% confidence intervals.

The predicted conditional MDPC is given in the panels of Figure 3. Figure 3A presents the pattern of MDPC (horizontal axis) across the levels of EQ (vertical axis) for after-tax income/month and the market value of a luxury car, while Figure 3B illustrates the pattern for the working hours/week and poverty rates (%) experiments. As can be seen, the conditional MDPC is highly heterogeneous for alternative levels of EQ both for “goods” and “bads.” Comparing confidence intervals across the levels of EQ unveils that MDPC for EQ levels from 45 to 65 are statistically significantly different from those MDPC for EQ levels below 40–45 for both “goods” (A) and “bads” (B). Among the unreported results, the standard errors obtained from the delta method are replaced with bootstrapped standard errors. The results hardly change. We also find a similar pattern in MDPC obtained from a non-parametric estimator, i.e., Spearman-Karber, and therefore the results are not presented in here.

### Robustness

#### Functional Form

First, we investigate the sensitivity of the baseline results (Figure 2) with respect to the functional form of EQ. The parameter estimates of the baseline model with the dummy indicating individuals with high EQ scores are presented in Row II of Table 2. The dummy for high EQ level is constructed by assigning a value of 1 for above-median EQ levels, EQ > 47, and zero for other levels. The signs and significance of the estimates are highly in line with those of the baseline. Next, we estimate a model with standardized values of EQ. In this specification, EQ enters the baseline specification (4) linearly and leads to highly similar results (Row III).

**Table 2:** Robustness: functional form, estimators, and omitted variables.

		**Pleasure and utility**			**Pain and disutility**		
		**Income (TRY/month)**	**Market value of a car (TRY)**	**I and II**	**Working hours (hours/week)**	**Poverty rate (% of people)**	**III and IV**
**Model specification**		**I**	**II**	**A**	**III**	**IV**	**B**
**Baseline (****Figure 2****)**							
I.	Log EQ	0.205^**^	0.150	0.178^**^	−0.181^*^	−0.288^***^	−0.226^***^
		(0.097)	(0.097)	(0.071)	(0.094)	(0.082)	(0.065)
	#Observations	224	214	438	231	231	462
**Functional forms**							
II.	High EQ dummy	0.129^***^	0.070	0.102^***^	−0.074^*^	−0.093^**^	−0.081^**^
		(0.046)	(0.045)	(0.033)	(0.042)	(0.046)	(0.032)
III.	Linear (standardized) EQ	0.050^**^	0.031	0.041^**^	−0.043^*^	−0.068^***^	−0.054^***^
		(0.024)	(0.024)	(0.018)	(0.024)	(0.021)	(0.017)
**Estimators**							
IV.	Linear model with OLS	0.228^**^	0.178^*^	0.202^***^	−0.186^*^	−0.272^***^	−0.197^***^
		(0.107)	(0.106)	(0.074)	(0.108)	(0.091)	(0.072)
V.	Probit	0.814^***^	0.659^**^	0.725^***^	−0.809^***^	−1.250^***^	−0.909^***^
		(0.240)	(0.259)	(0.175)	(0.248)	(0.243)	(0.171)
VI.	Ordered Probit	0.740^*^	0.753^*^	0.707^**^	−0.695^*^	−1.221^***^	−0.887^***^
		(0.378)	(0.424)	(0.280)	(0.402)	(0.381)	(0.278)
**Further proxies for omitted variables**							
VII	Prosocial behavior	0.195^**^	0.144	0.170^**^	−0.186^**^	−0.281^***^	−0.222^***^
		(0.096)	(0.097)	(0.071)	(0.094)	(0.083)	(0.066)
VIII.	Competitivity	0.209^**^	0.149	0.182^***^	−0.182^*^	−0.285^***^	−0.225^***^
		(0.095)	(0.096)	(0.070)	(0.094)	(0.082)	(0.065)
	#Observations	672	642	1,314	693	693	1,386

*Author's own calculations from the experimental data*.

*The models allow for the full set of control variables (see Figure 2)*.

*(A) combines the income and car experiments while (B) combines the working hours and poverty rates experiments*.

*Robust standard errors are presented in the parentheses*.

^*^, ^**^, and ^***^ indicate significance level at 10, 5, and 1% levels of significance, respectively.

#### Estimators

The model specification in Equation (4) is also estimated with alternative estimators. First is the ordinary least squares (OLS) estimator, where the dependent variable is redefined as the midpoints, e.g., (0 + 0.25)/2 for the first interval and so on, of each marginal positionality interval. The parameter estimates presented in Row IV are highly similar to those from the baseline specification (Row I). However, unlike the baseline interval regressions, OLS produces statistically significant estimates for all goods and their combinations. Second, the dependent variable is redefined as a dummy variable indicating the positional choice (Society B) in any choice situation for each good. The model specification is then a binary choice model and is estimated with the probit model. The results presented in Row V indicate highly similar with more precise parameter estimates^11^. Third, an ordered probit model is estimated by assigning ordinal values for the marginal positionality intervals as ${\tilde{\theta }}_{i}^{g}=0,\;1,\;2,\;3$ for each individual *i* and good *g*. This model specification is only slightly different from the baseline interval regression. It assumes that the cut-off points for the marginal positionality intervals are unknown constants and they are simultaneously estimated within the same estimation process. The parameter estimates are presented in Row VI. They are all statistically significant and have the same signs and significance levels of those found in the baseline (Row I).

#### Further Omitted Variables

To deal with bias due to endogeneity generated by omitted variables, we experiment with further proxies that might be correlated with EQ and error terms. Two key variables that we focus on are prosocial behavior (e.g., helping behavior, altruism, or cooperation) and degree of competitivity. Recent literature identifies an important positive relationship between empathy and prosocial behavior, while there is an opposite relationship between empathy and competitivity (e.g., Klimecki et al., 2016). To identify the degree of prosocial behavior, we elicit a detailed measure for the helping or volunteering behavior of respondents, which might also be a measure of their degree of altruistic behavior. The respondents were asked whether they had taken part in any volunteer activities in the past year (see Appendix C.2 for the full set of volunteer activities). The measure is created by simply summing up the binary responses to all volunteering items. Implementing the measure in our baseline interval regression hardly changes any estimation results (Row VII of Table 2).

Then, we elicited a proxy for the degree of competitivity using three questions (see Appendix C.3 for the full set of questions). The questions aim to elicit the desire of respondents living in “*egalitarian-competitive*,” “*welfare state-individualistic*,” and “*regulated-deregulated societies*.” Each question is responded from 1 to 5 as 1 “*closer to first*,” 2 “*somewhat closer to first*,” 3 “*can't say which*,” 4 “*somewhat closer to second*,” and 5 “*closer to second*.” To determine the proxy for the degree of competitiveness, we sum the answers to the three questions. The proxy is then controlled for in the baseline regression and the results are presented in Row VIII of Table 2. The parameter estimates are highly similar to those obtained from the baseline^12^.

## Evidence from Subjective Well-Being Data

Another approach to investigate positional concern is based on SWB regressions (Ferrer-i-Carbonell, 2005; Luttmer, 2005; Senik, 2005; Clark et al., 2008; Akay and Martinsson, 2011). In these regressions, SWB, e.g., life satisfaction or happiness, is used as a proxy for (experienced) utility (Kahneman and Sugden, 2006)^13^. Then SWB regressions are estimated on own level of consumption of a good and on a reference (or comparison) level of consumption by others, i.e., a reference group. The literature aiming to identify positional concerns using SWB datasets has grown rapidly in recent years (e.g., see Clark et al., 2008 for a comprehensive review). The literature reports that SWB is negatively affected by income comparisons in developed countries (Ferrer-i-Carbonell, 2005; Luttmer, 2005), but positively affected or not significantly affected in transition (Senik, 2004) and developing countries (Akay and Martinsson, 2011). In this section, we present complementary evidence based on a large survey that includes data on, e.g., SWB, degree of empathic capacity, absolute and reference per capita after-tax income/month, and working hours/week.

### Data

The dataset at use is the General Social Survey (GSS), which is a large and nationally representative cross-sectional dataset collected since 1978^14^. It is very rich with respect to socio-demographic and -economic characteristics and includes a wealth of subjective opinion questions, e.g., attitudes to empathy and a large list of proxies for prosocial behavior. Our sample selection is straightforward. In our analysis, we use people older than 17 and younger than 75 years of age. The empathy information is available in the 2002 and 2004 waves in the National Altruism Study Module which is a part of the GSS dataset. Having deleted the missing values in all variables used in our analysis leaves a sample size of 2,237 individuals. The SWB measure is based on “happiness” information about individuals obtained by means of the following question: “*Taken all together, how would you say things are these days – would you say that you are very happy, pretty happy, or not too happy*?” The variable is observed on 3-point ordinal scale that aims to capture the respondent's subjective welfare experience. In our SWB regressions, we allow for a large set of individual socio-demographic and -economic characteristics that are often used in well-being regressions (see, e.g., Dolan et al., 2008 for a comprehensive review).

### Measures

#### Measure of Empathy

The dataset allows us to calculate (Davis, 1980, 1983) interpersonal reactivity index (IRI). This measure is based on responses to seven expressions/statements, e.g., “*I often have tender, concerned feelings for people less fortunate than me*” and “*I am often quite touched by things that I see happen*” (see Appendix D.1 for the full set of statements) on a 5-point scale, where 1 = “*completely disagree*” and 5 = “*completely agree*” for items (1), (3), (6), and (7) and the opposite for items (2), (4), and (5). Then we simply calculate the average score for the seven items. In line with the EQ measure of empathy, a higher IRI indicates a higher degree of dispositional or trait empathy. The mean IRI is 3.94 (std. 1.24). The Cronbach's alpha for the seven-items of IRI inventory and for all respondents in the GSS data is 0.73, which indicates a relatively high internal consistency.

#### Consumption Goods and Their Absolute and Relative Levels

To sustain comparability with the experimental results, we investigate two consumption items that are highly in line with those used in our experiments: per capita after-tax income/month as a “good” and working hour/week as a “bad.” After-tax income is the total after-tax family income from all sources in a year divided by 12. To obtain the *per capita* after-tax income/month, we use weights of the standard OECD equivalence scale (1 for the individual, 0.7 for each adult, and 0.5 for each child in the household). To obtain average weekly working hours, we use the average hours spent on the primary job for each individual. We simply use zero working hours for those who were unemployed in the previous survey year.

To measure relative levels of per capita after-tax income/month and working hours/week, the reference groups with which individuals compare their income or working hours should be defined. As in the bulk of the SWB literature, our approach is based on defining reference groups using some criteria, e.g., age, gender, and region (e.g., Clark and Oswald, 1996; Ferrer-i-Carbonell, 2005; Luttmer, 2005). Recent studies also show that defining reference groups with *ad-hoc* criteria and directly asking individuals about their reference group produce highly similar results (Clark and Senik, 2010). The reference groups that we use are based on age, gender, health status, marital status, and region of residence. We use combinations of these criteria for each reference group used in our estimations. Our baseline reference group definition suggests that “*the individuals compare their per capita after-tax household income (working hours/week) with the average per capita after-tax household income (working hours/week) of all people who live in the same region (nine regions), who are in the same age group (four quartiles of age distribution), and who are of the same gender (male or female)*.” The number of reference groups with this definition is 72, each consisting of about 30 individuals. We then use the average per capita after-tax family income/month or average working hours/week of the reference group as the reference income or reference working hours with which the individuals compare their own income or own hours of work. Next, we add marital status (married = 1) and health status (very good health = 1) in the definition to check the robustness of the results.

### Econometric Approach and Results

#### Model Specification

To investigate how positional concerns are heterogeneous with respect to the degree of empathic capacity, we are going to estimate a series of well-being equations. SWB is measured on a 3-point ordinal scale and the appropriate model is an ordinal choice model. The baseline model specification, in which we estimate the absolute and reference consumption levels on SWB for a good, is as follows:

(5) $$\begin{array}{r} SWB_{i}^{*}=\lambda_{A}ln\left(Y_{i}^{A}\right)+\lambda_{R}ln\left(Y_{r}^{R}\right)+{\text{X}}^{\prime }\beta +s_{k}+\tau_{t}+\epsilon_{i}. \end{array}$$

In Equation (5), *SWB*_*i*_ is the happiness measure and takes the values of *J* = 1, 2, 3, and *i* indicates the individual. $Y_{i}^{A}\;$is the own level of per capita after-tax income or own working hours/week. $Y_{r}^{R}$ is the reference level of per capita after-tax income/month or working hours/week and is calculated as $Y_{r}^{R}=\left(1/N_{r-1}\right)\overset{N_{r-1}}{\underset{m=1}{\sum }}Y_{m}^{R}$, which is the “average” level of per capita after-tax income/month or working hours/week in individual *i*'s reference group *r*. *N*_*r*_ is the number of people in the reference group^15^.

λ_*A*_ is the parameter of own consumption while λ_*R*_ is the parameter for the reference consumption, which is a measure of the positional concerns as it indicates the strength of the relationship between the consumption level of people in the reference group and individuals' well-being. The sign of λ_*A*_ is expected to be positive for after-tax income/month as a higher level of resources implies a higher level of well-being. The literature suggests that time spent on working is associated with disutility, implying a negative relationship between own working hours and well-being (Knabe and Rätzel, 2010; Rätzel, 2012). Yet longer working hours also implies a higher level of resources, which might correlate positively with well-being. Thus, the sign of the relationship between own working hours on well-being is a priori unknown. λ_*R*_ is expected to be *negative* for per capita after-tax income/month and *positive* for working hours. While a higher level of income of others implies a lower income position, a higher level of working hours among others implies a higher level of indirect benefits for the individual.

The main aim of this section is to investigate how λ_*R*_ varies with respect to the degree of empathic capacity, IRI. To this end, interaction models are used. λ_*R*_ in the model specification (5) is replaced with ${\lambda_{R}\;=\lambda_{R}^{LIRI}D}_{i}+\lambda_{R}^{HIRI}\left(1-D_{i}\right)$, where *D*_*i*_ is a dummy variable indicating individuals with high IRI levels. We define high levels of empathic capacity using the median IRI = 4 as threshold. The hypothesis we test is whether $\lambda_{R}^{LIRI}$ is equal to $\lambda_{R}^{HIRI}$ for per capita after-tax income/month and working hours/week in separate regressions.

#### Specifications

The model specification allows for a large set of individual and household characteristics, **X**, including age, gender, health status (in four dummies from “very poor” to “very good”), years of education, marital status (dummies for married, single, widowed, and divorced), number of children at home (dummies for kids 1–5, 6–11, and 12–17 years old), total household size, race (dummies for white, black, and other), labor market status (dummies for working full-time, working part-time, temporarily not working, retired, and in school). β is a vector of parameters corresponding to the control variables in matrix *X*. The model also allows for nine region^16^ dummies *s*_*k*_. The model specification pools data from two waves and τ_*t*_ is the dummy for the 2004 wave. ϵ_*i*_ is the usual error term.

An appropriate model specification for Equation (5) is an ordered probit, which exploits the ordinal nature of the dependent variable. Yet, recent research shows that there is basically no difference between a linear model and ordered probit specification (Ferrer-i-Carbonell and Frijters, 2004). To exploit the simplicity of linear models, we prefer ordinary least squares as our baseline model specification. However, we also estimate models with the ordered probit model specification and compare the results.

### Results

#### Main Results

We estimate the well-being regression in (5) with and without the interaction terms for two alternative goods, i.e., per capita after-tax income/month and working hours/week. The results are summarized in Table 3^17^. First, we estimate the baseline model specification (5), where we allow only for absolute and reference income without interaction terms (Column I). In line with the expectations, the absolute level of income is positively related to happiness while the reference income is negatively associated. These results are also highly in line with the literature (Ferrer-i-Carbonell, 2005; Luttmer, 2005). The significant relationship between reference income and happiness is an indicator of a degree of positional concern. Our main aim is to test whether the relationship between reference income and happiness is heterogeneous with respect to the degree of empathic capacity measured by IRI. The results from the baseline interaction model are given in Column II of Table 3. There is substantial heterogeneity in estimated reference income (Rows A and B). The reference income on SWB is negative and statistically significant only among high-IRI people. The difference between parameter estimates for low and high degree of empathic capacity is statistically significant at the conventional levels of significance (*p* = 0.068). That is, a higher level of empathic capacity is associated with a stronger negative effect of positional concerns regarding per capita after-tax income/month on happiness. This result is highly consistent with the results from our survey experiment above.

**Table 3:** Results from subjective well-being approach.

	**Real family income per capita**					**Average weekly working hours**				
	**Baselines**		**Robustness**			**Baselines**		**Robustness**		
		**Interaction model**	**RG-1**	**RG-2**	**Prosocial behavior**		**Interaction model**	**RG-1**	**RG-2**	**Prosocial behavior**
	**I**	**II**	**III**	**IV**	**V**	**VI**	**VII**	**VIII**	**IX**	**X**
IRI measure of empathy	0.020					0.020				
	(0.020)					(0.020)				
Absolute level	0.045^***^	0.044^***^	0.045^***^	0.045^***^	0.044^***^	0.030	0.026	0.026	0.020	0.031
	(0.016)	(0.016)	(0.016)	(0.015)	(0.016)	(0.058)	(0.058)	(0.061)	(0.052)	(0.058)
Relative level	−0.201^***^					0.064^*^				
	(0.076)					(0.037)				
High IRI (= 1 if greater than median = 4)		−2.045^*^	−1.255^*^	−1.372^**^	−1.710^*^		−0.141^*^	−0.136^*^	−0.137^*^	−0.171^*^
		(1.110)	(0.747)	(0.683)	(0.979)		(0.082)	(0.076)	(0.077)	(0.098)
A. Relative Level ^*^ Low IRI		−0.118	−0.129	0.001	−0.099		0.096^*^	0.092^**^	0.097^**^	0.110^*^
		(0.108)	(0.082)	(0.069)	(0.100)		(0.053)	(0.046)	(0.048)	(0.057)
B. Relative Level ^*^ High IRI		−0.324^***^	−0.259^***^	−0.140^***^	−0.274^***^		0.017	0.015	0.020	0.033
		(0.082)	(0.081)	(0.061)	(0.083)		(0.048)	(0.045)	(0.045)	(0.050)
*P*-value (H0: A = B)		0.0675	0.084	0.041	0.0772		0.0188	0.013	0.018	0.0462
*R*-Squared	0.149	0.166	0.151	0.15	0.151	0.15	0.152	0.152	0.151	0.166
#Observations	2,237	2,237	2,237	2,237	2,237	2,237	2,237	2,237	2,237	2,237

*Authors' own calculations from GSS (2002 and 2004)*.

*The dependent variable is the happiness which is measured in 3-point scale. The models are estimated with ordinary least squares. The control variables include age, gender, health status (in four dummies from “very poor” to “very good”), years of education, marital status (dummies for married, single, widowed and divorced), number of children at home (dummies for kids 1–5, 6–11, and 12–18 years old), total household size, race (dummies for white, black, and other), labor market status (dummies for working full-time, working part-time, temporary not working, retired, and in school). The model also allows for nine regional dummies*.

*To calculate per capita household income, the standard OECD scale is used*.

*The standard errors are clustered at the reference groups level*.

*List of measures for prosocial behavior is in Appendix D.2*.

^*^, ^**^, and ^***^ indicate significance at 10, 5, and 1%, respectively.

Next, we turn our attention to positional concerns regarding working hours/week and conduct a similar analysis as for per capita after-tax income/month. The results from the baseline model (5) without the interaction terms are presented in Column V of Table 3. The absolute working hour/week on SWB is statistically insignificant while the reference working hours/week on happiness is positive and statistically significant, which is also in line with the expectations. We estimate the baseline model with interaction terms and present the results in Column VI. The results are strikingly consistent with those from the survey experiment. A higher level of empathic capacity leads to a weaker and statistically insignificant relationship between reference working hours/week and SWB, implying a degree of other-regarding feelings or behavior. The parameter estimate of reference working hours is large, positive, and statistically significant among people with a lower level of empathy. The difference between the parameter estimates of reference working hours/week by low and high empathy is also statistically significant (*p* = 0.019).

#### Robustness

We extensively investigate the robustness of the baseline results and summarize our findings in Table 3. Our robustness testing presented here mainly targets the definition of reference groups and potential omitted variables, which are the key threats to the estimation results. Two additional criteria are added in the baseline definition of the reference groups. First, a dummy for married individuals (married = 1) is used together with age (four quartiles of the age distribution), gender, and regions (nine regions) in RG-1^18^. The results from RG-1 are given in Columns III and VIII for per capita after-tax income/month and working hours/week, respectively. Adding marital status into the reference group definition does not substantially affect the parameter estimates and test results. In RG-2 (Columns IV and IX), we add health status into the baseline definition of the reference group. The health status is defined using a dummy variable indicating individuals with “good” and “very good” health. The size of the reference income estimates with RG-2 is somehow reduced for the per-capita after-tax income/month. Yet the differences across the low and high levels of IRI are still statistically significant (*p* = 0.041). The results for working hours/week are highly similar to those of the baseline (Column VI).

Finally, we investigate the robustness with respect to potential omitted variables. As our dataset is cross-sectional, we are not able to allow for unobserved individual effects (e.g., typically considered to be personality dispositions or genetic factors). If these characteristics are correlated with IRI, the results presented in Table 3 might be biased. As in the case of our survey experiment, we control Equation (5) for some proxy variables that may be correlated with EQ and error terms. The dataset includes a rich set of variables that can be used for this purpose. In line with the previous analysis, we mainly focus on the prosocial behavior measured using attitudes to altruism (e.g., volunteering and helping behavior). The measures are obtained using the set of 15 questions in the National Altruism Study Module of GSS dataset supplied for the years 2002 and 2004. The full set of questions in this module is given in Appendix D.2 (see also Einolf, 2008, for further discussions).

Our modeling strategy is to include these 15 proxies for altruistic attitudes and helping behavior in our baseline interaction model specification and check whether the previous results stay the same. These results are presented in Columns V and X of Table 3. Adding these variables have only a marginal influence on the parameter estimates and the test results. The differences in the parameter estimates of reference per capita after-tax income/month and working hour/week on SWB for low and high IRI are still statistically significant. Among the unreported results, we conducted several further sensitivity checks. First, we experimented by creating alternative proxies by summing or averaging all items in Appendix D.2. The results are practically the same. Second, we estimated our interaction models with an alternative set of control variables and estimators. We estimated the baseline interaction model using a stepwise estimation strategy and also with the ordered probit model specification. The results presented in Table 3 hardly changed.

## Concluding Discussions

Empathic capacity measured using both the empathy quotient (Baron-Cohen and Wheelwright, 2004) and the interpersonal reactivity index (Davis, 1983) is significantly associated with positional concerns identified using survey experiments and the subjective well-being (SWB) approach. The experiments were conducted for an alternative set of goods associated with individual pleasure and suffering to investigate how people's levels of empathy relate their positional concerns with regard to a number of consumption “goods” and “bads.” The SWB approach investigates how the utility impact of others' consumption is heterogenous with respect to the level of empathic capacity. Our main conclusion is that positional concerns substantially vary with the levels of empathic capacity. The degree of heterogeneity in positional concerns differ across types of goods in a predictable pattern. The results are very robust and suggest that people with a higher level of empathic capacity are more concerned about their relative consumption position (or their utility is affected more) when the object of their comparisons is a consumption item associated with pleasure and utility while they are less positional (or their utility is affected less) when it is a consumption item associated with pain and disutility. Extensive robustness analysis suggests that the results are insensitive with respect to functional forms, estimators, empathy measure used, and potential omitted variables (e.g., prosocial behavior) that may bias the results.

Our results are highly intuitive and suggest that positional concerns vary with empathic capacity. One obvious practical implication of this finding for economics is that the models aiming to relate optimal taxation, labor supply, and consumption decisions with positional concerns should also consider the heterogeneity in positional concerns due to dispositional empathy differences across individuals. However, caution should be taken. In conceptual terms, we rely on the definition of a trait-like empathy, i.e., dispositional empathic capacity. Thus, the results in this paper can be interpreted as part of a recently developing literature aiming to investigate how non-cognitive skills relate to economic outcomes of individuals (e.g., Borghans et al., 2008). However, empathy can also change temporally depending on contextual factors, and the utility implications of these temporal changes might depend on another set of factors, e.g., the speed of adaptation to temporal shocks. Our first suggestion for future research is to extend the research presented here to experiments where temporal empathy is measured (see, e.g., Klimecki et al., 2016). Two important limitations of this study are that the experiment uses student respondents and assumes a fixed composition of individuals in the reference groups. Second suggestion for the future research is to use more representative sample of individuals where potential life-cycle changes in personality characteristics are identified. As the psychology literature suggests, people's empathic reflection process may also differ depending on the socio-cultural or genetic proximity to the people in their reference groups (Brandstätter, 2000). In our paper, we assumed that individuals' reference groups are exogenously given and formed by “strangers.” Thus, our final suggestion for future research is to investigate how the characteristics of people in the reference group interfere with the relationship between empathy and positional concerns.

## Data Availability Statement

The datasets generated for this study are available on request to the corresponding author.

## Ethics Statement

According to the Turkish law, the experiment did not require an ethical committee approval and also there was no institutional review board for the social sciences in the Istanbul University by the time of our experiment, 2014. Therefore, written informed consent was not obtained from participants. Students are recruited. The paper includes detailed information on the recruitment process.

## Author Contributions

All authors listed have made a substantial, direct and intellectual contribution to the work, and approved it for publication.

### Conflict of Interest

The authors declare that the research was conducted in the absence of any commercial or financial relationships that could be construed as a potential conflict of interest.

## Footnotes

^1^The term empathy was not yet available when Adam Smith discussed the relationship between “sympathy” and non-selfish behavior. He used the term sympathy almost synonymously to the current meaning of empathy. In recent literature, sympathy is considered an “affective” component of empathy (de Waal, 2008). See Fontaine (2001) and Sugden (2001) for historical accounts of the terms.

^2^Different strands of literature from a wide variety of disciplines have investigated the evolutionary, neurobiological, and genetic roots of affective and cognitive dimensions of empathy (de Waal, 2008, 2012; Preckel et al., 2018; Blagrove et al., 2019). In studies involving humans and animals, neurobiologists identify mirror-neurons that operate during empathic processes (e.g., Rizzolatti and Craighero, 2004; Fogassi, 2011; Khalil, 2011; Molnar-Szakacs, 2011; Bernhardt and Singer, 2012; Cronin, 2012).

^3^The term good should be clarified. We use it to mean any tangible or intangible commodity. To differentiate between goods associated with pleasure/utility and pain/disutility, we use the terms “goods” and “bads” (always in quotation marks), respectively.

^4^According to the Turkish law, the experiment did not require an ethical committee approval and also there was no institutional review board for the social sciences in the Istanbul University by the time of our experiment, 2014. A written consent was not obtained from participants. Students voluntarily registered for the experiment and consents of the participants was implied through survey completion.

^5^The respondents were recruited from three departments, economics, psychology, and law, of Istanbul University, Turkey. We announced the experiment with a poster on the boards of the student hall of each department. The experiment was conducted among the voluntary participants in three sessions in a large lecture hall. At the beginning of the experiment, the students were also told to feel free to leave the experiment anytime. The respondents were guided by five experimental assistants who presented the scenario of the experiment and answered any questions asked by the respondents. The experimental sessions lasted about an hour and the respondents were given a supplementary textbook that was priced about the average hourly wage in Istanbul at the time of the experiment in 2014.

^6^Some respondents make choices that are inconsistent with the utility maximization assumption. That is, the utility maximization assumption predicts that once a respondent chooses Society (A), she should not choose the positional alternative Society (B) for a larger implicit degree of positional concerns. We identified these respondents and simply removed them from the sample used in our analysis below. The share of inconsistent respondents is about 10–15% across the goods.

^7^To measure the degree of inequality aversion, we elicit subjective attitudes to inequality using the questions as follows. Using a 1–7 scale, the participants reported their preference regarding two sets of statements, i.e., (A) “income should be more equal as incentives” (1) vs. “we need larger income differences for higher effort” (7) and (B) “an egalitarian society where the gap between rich and poor is small, regardless of achievement” (1) vs. “a society, where wealth is distributed according to ones' achievement” (7). Then we obtained the measure of subjective inequality aversion by simply adding the two scores reported for (A) and (B).

^8^The Big-5 is measured based on 15 questions obtained from the 2009 questionnaire of the German Socio-Economic Panel. See http://www.diw.de for further information.

^9^One important remark is that, in our baseline model specification, the first (last) boundaries of the marginal positionality intervals are assumed to be censored below (above). We also estimate models by assuming 0 and 1 for the censored boundaries. The results are practically the same.

^10^The full estimation results are not presented due to space reasons, but can be provided by the authors upon request.

^11^We present the parameter estimates instead of marginal effects as we are mainly interested in comparing the signs and significance of these estimators with those of the baseline. The marginal effects can be reported upon request.

^12^Among the unreported results, we also allow our regressions for “dispositional envy” and “self-esteem” in separate regressions. Envy is often considered an emotion underlying positional behavior. To measure envy, we use the Dispositional Envy Scale developed by Smith et al. (1999). Introducing dispositional envy only slightly increases the magnitude of estimates for the “goods,” while there is practically no effect on the parameter estimates for “bads.” Finally, we use the Rosenberg (1985) inventory for “self-esteem.” The literature suggests that self-esteem is related to both empathy and positional behavior for several goods including physical appearance or career success (e.g., Vrabel et al., 2018). Adding the self-esteem measure in the baseline slightly increased the estimates. Importantly, in the specification with self-esteem, the EQ on positional concern is statistically significant for all consumption items including the market value of a luxury car.

^13^Several studies have validated SWB measures as a measure of well-being (Krueger and Schkade, 2008). Today there is a consensus that these simple subjective questions can indeed capture levels of individual welfare (e.g., Oswald and Wu, 2010).

^14^The data are collected by the National Opinion Research Center at the University of Chicago. The dataset is obtained from http://gss.norc.org/get-the-data. Please visit the website for further information on the sampling frame and measures.

^15^As in the bulk of the literature investigating positional concerns, we also use the parameter estimates of the reference income as a measure of the degree of positional concerns (e.g., Ferrer-i-Carbonell, 2005). Another strategy to identify positional concerns is to use the $log\left(Y_{i}^{A}/Y_{r}^{R}\right)$*, w*hich is practically the same as the specification in (5).

^16^The dataset does not include information on the federal states where the individuals reside. Instead, we use a regional classification based on nine groups of federal states: New England, middle Atlantic, south Atlantic, east and west north central, east and west south central, mountain, and Pacific states.

^17^The full estimation results of the well-being regressions are not reported here due to space limitations. However, they are highly similar to those in the literature. Age and happiness have a U-shaped relationship while health and employment status are positively related to happiness. All estimation results are available upon request from the authors (see Dolan et al., 2008).

^18^Note that the precision of reference income is highly related to the size of reference groups. Thus, adding more criteria substantially reduces both the sample size and the reference income and working hours estimates.
