# Supplementary material for: Standing in Others' Shoes: Empathy and Positional Behavior
Source: Front Psychol. 2019 Oct 17;10:2226. doi: 10.3389/fpsyg.2019.02226 (PMC6811653; doi:10.3389/fpsyg.2019.02226)
Supplement: Supplementary file 1 [file Data_Sheet_1.docx]

**Online Appendices**

**Appendix A. The Scenario and Example Choice Situation**

**Appendix B.1. The Survey Experiment (After-Tax Income/Month)**

| Income of your future relative (1)  In the situation below, make a choice between society A and society B for your future relative.  Society A: – Your relative’s income is 2000 TRY/month after tax  – The average income in society is 2200 TRY/month after tax  Society B: – Your relative’s income is 1800 TRY/month after tax  – The average income in society is 1400 TRY/month after tax  Everything, including the price levels, are same in two societies. In both societies your relative works 40 hours per week and this is equal to the average weekly work hours. Choose the society in which your future relative will be most content.   \| Society A \| Society B \| \| --- \| --- \| \|  \|  \| |
| --- | --- | --- | --- | --- |

| Income of your future relative (2)  In the situation below, make a choice between society A and society B for your future relative.  Society A: – Your relative’s income is 2000 TRY/month after tax  – The average income in society is 2200 TRY/month after tax  Society B: – Your relative’s income is 1550 TRY/month after tax  – The average income in society is 1300 TRY/month after tax  Everything, including the price levels, are same in two societies. In both societies your relative works 40 hours per week and this is equal to the average weekly work hours. Choose the society in which your future relative will be most content.   \| Society A \| Society B \| \| --- \| --- \| \|  \|  \| |
| --- | --- | --- | --- | --- |

| Income of your future relative (3)  In the situation below, make a choice between society A and society B for your future relative.  Society A: – Your relative’s income is 2000 TRY/month after tax  – The average income in society is 2200 TRY/month after tax  Society B: – Your relative’s income is 1220 TRY/month after tax  – The average income in society is 1160 TRY/month after tax  Everything, including the price levels, are same in two societies. In both societies your relative works 40 hours per week and this is equal to the average weekly work hours. Choose the society in which your future relative will be most content.   \| Society A \| Society B \| \| --- \| --- \| \|  \|  \| |
| --- | --- | --- | --- | --- |

**Appendix B.2. The Summary of Survey Experiment**

**Appendix C.1. Empathy Quotient (Baron-Cohen and Wheelwright, 2004)**

Here indicate how closely you agree with the statement by checking one of the boxes on the scale between 1 to 4. 1 means strongly agree, and 4 means you strongly disagree. If your views fall somewhere in between, you can choose any number in between. The control/filter questions are marked with italics.

1. I can easily tell if someone else wants to enter a conversation.
2. I prefer animals to humans.
3. I try to keep up with the current trends and fashions.
4. I find it difficult to explain to others things that I understand easily, when they don’t understand it first time.
5. I dream most nights.
6. I really enjoy caring for other people.
7. I try to solve my own problems rather than strongly discussing them with others.
8. I find it hard to know what to do in a social situation.
9. I am at my best first thing in the morning.
10. People often tell me that I went too far in driving my point home in a discussion.
11. It doesn’t bother me too much if I am late meeting a friend.
12. Friendships and relationships are just too difficult, so I tend not to bother with them
13. I would never break a law, no matter how minor.
14. I often find it difficult to judge if something is rude or polite.
15. In a conversation, I tend to focus on my own thoughts rather than on what my listener might be thinking.
16. I prefer practical jokes to verbal humor.
17. I live life for today rather than the future.
18. When I was a child, I enjoyed cutting up worms to see what would happen.
19. I can pick up quickly if someone says one thing but means another.
20. I tend to have very strong opinions about morality.
21. It is hard for me to see why some things upset people so much.
22. I find it easy to put myself in somebody else’s shoes.
23. I think that good manners are the most important thing a parent can teach their child.
24. I like to do things on the spur of the moment.
25. I am good at predicting how someone will feel.
26. I am quick to spot when someone in a group is feeling awkward or uncomfortable.
27. If I say something that someone else is offended by, I think that that’s their problem, not mine.
28. If anyone asked me if I liked their haircut, I would reply truthfully, even if I didn’t like it.
29. I can’t always see why someone should have felt offended by a remark.
30. People often tell me that I am very unpredictable.
31. I enjoy being the center of attention at any social gathering.
32. Seeing people cry doesn’t really upset me.
33. I enjoy having discussions about politics.
34. I am very blunt, which some people take to be rudeness, even though this is unintentional.
35. I don’t tend to find social situations confusing.
36. Other people tell me I am good at understanding how they are feeling and what they are thinking.
37. When I talk to people, I tend to talk about their experiences rather than my own.
38. It upsets me to see an animal in pain.
39. I am able to make decisions without being influenced by people’s feelings.
40. I can’t relax until I have done everything I had planned to do that day.
41. I can easily tell if someone else is interested or bored with what I am saying.
42. I get upset if I see people suffering on news programmes.
43. Friends usually talk to me about their problems as they say that I am very understanding.
44. I can sense if I am intruding, even if the other person doesn’t tell me.
45. I often start new hobbies but quickly become bored with them and move on to something else.
46. People sometimes tell me that I have gone too far with teasing.
47. I would be too nervous to go on a big rollercoaster.
48. Other people, often say that I am insensitive, though I don’t always see why.
49. If I see a stranger in a group, I think that it is up to them to make an effort to join in.
50. I usually stay emotionally detached when watching a film.
51. I like to be very organized in day-to-day life and often make lists of the chores I have to do.
52. I can tune into how someone else feels rapidly and intuitively.
53. I don’t like to take risks.
54. I can easily work out what another person might want to talk about.
55. I can tell if someone is masking their true emotion.
56. Before making a decision I always weigh up the pros and cons.
57. I don’t consciously work out the rules of social situations.
58. I am good at predicting what someone will do.
59. I tend to get emotionally involved with a friend’s problems.
60. I can usually appreciate the other person’s viewpoint, even if I don’t agree with it.

**Appendix C.2. Helping Behavior (Volunteering)**

Have you done any volunteer work (other than housework, unpaid) in the last 12 months? Indicate by checking “Yes” to the item(s) that describes best the type of your volunteer activity. Check mark “No” if the item is not suitable. The scale is created by summing up to answers to all questions.

| The type of the volunteer work |
| --- |
| Unpaid public work |
| Cleaning, repairing and construction |
| Youth organizations, youth clubs, scouting |
| Activities and/or meetings of organizations |
| Looking after the children and/or people of other families |
| Other help to other families |
| Charity organization that helps the seniors, the disabled, and the poor |
| Public education activities |
| Cultural and/or other public activity organizations |
| Religion organizations, associations, mosque-building associations etc. |
| Organizations working on environment, animal rights etc. |
| Raising money for volunteer organizations |
| Other volunteer activities |

**Appendix C.3. Competitivity**

Which type of society do you think a country should aim to be in the future? For each pair of statements, would you prefer being closer to the first or to the second alternative?

| *An egalitarian society where the gap between rich and poor is small, regardless of achievement* | | | | *A competitive society, where wealth is distributed according to ones’ achievement* | | | |
| --- | --- | --- | --- | --- | --- | --- | --- |
|  | Closer to  first | Somewhat closer to first | Can’t say which | | Somewhat closer to second | Closer to second |  |
| *A society with extensive social*  *welfare, but high taxes* | | | | *A society where taxes are low and individuals*  *take responsibility for themselves* | | | |
|  | Closer to  first | Somewhat closer to first | Can’t say which | | Somewhat closer to second | Closer to second |  |
| *A society that assures safety and stability*  *through appropriate regulations* | | | | *A deregulated society where people are*  *responsible for their own actions* | | | |
|  | Closer to  first | Somewhat closer to first | Can’t say which | | Somewhat closer to second | Closer to second |  |

**Appendix D.1. Interpersonal Reactivity Index (Davis, 1983)**

The following statements ask about your thoughts and feelings in various situations. For each item indicate how well it describes you by choosing the number on the show card where 1 indicates that it does not describe you very well and 5 means that it does describe you very well. Of course, numbers 2–4 indicate that how well it describes you are in between these points.

(1) I often have tender, concerned feelings for people less fortunate than me

(2) Sometimes I don’t feel very sorry for other people when they are having problems

(3) When I see someone being taken advantage of, I feel kind of protective towards them

(4) Other people’s misfortunes do not usually disturb me a great deal

(5) When I see someone being treated unfairly, I sometimes don’t feel very much pity for them

(6) I am often quite touched by things that I see happen

(7) I would describe myself as a pretty soft-hearted person

To obtain the IRE empathy scale, we first reverse the items 2, 4, and 5, and then average the scores of these seven items.

**Appendix D.2. Helping behaviors (General Social Survey, Topical Module on Altruism, 2002 and 2004)**

During the past 12 months, how often have you done each of the following things:

(1) Donated blood

(2) Given food or money to a homeless person

(3) Returned money to a cashier after getting too much change [this question was not used in this study]

(4) Allowed a stranger to go ahead of you in line

(5) Done volunteer work for a charity

(6) Given money to a charity

(7) Offered your seat on a bus or in a public place to a stranger who was standing

(8) Looked after a person’s plants, mail, or pets while they were away

(9) Carried a stranger’s belongings, like groceries, a suitcase, or shopping bag

(10) Given directions to a stranger

(11) Let someone you didn’t know well borrow a item of some value like dishes or tools

During the past 12 months, how often have you done any of the following things for people you know personally, such as relatives, friends, neighbors or other acquaintances?

(12) Helped someone outside of your household with housework or shopping

(13) Lent quite a bit of money to another person

(14) Spent time talking with someone who was a bit down or depressed

(15) Helped somebody to find a job
